# A multifaceted approach in reducing central line associated bloodstream infections (CLABSI) in pediatric icus at a tertiary hospital

**DOI:** 10.1186/2047-2994-4-S1-P213

**Published:** 2015-06-16

**Authors:** H Balkhy, AM Al Shehri, NL Dagunton, N Dagunton

**Affiliations:** 1 Infection Prevention & Control Department, King Abdulaziz Medical City for Ministry of National Guard, Riyadh, Saudi Arabia

## Introduction

Studies have shown that collaborative efforts of multidisciplinary teams led to the reduction of CLABSI rate. However, no published studies had been reported from the Kingdom of Saudi Arabia (KSA) which examined the impact of team approach in reducing CLABSI rates. We examined CLABSI rate at the 20-bed Pediatric Medical/Surgical intensive care unit (PICU) at King Abdulaziz Medical City (KAMC), Riyadh, KSA before and after collaborative efforts.

## Objectives

Our interventions were directed at reducing CLABSI rate to zero.

## Methods

Using the guidelines from the Institute of Healthcare Improvement (IHI) and the National Healthcare Safety Network (NHSN), a 24-month prospective surveillance was conducted from the 2^nd^ quarter of 2010 to the 1^st^ quarter of 2013.

Central line insertion bundle was initiated throughout the study period. The PICU formed a CLABSI team during the 3rd quarter of 2012 through a multidisciplinary collaborative team approach composed of nurses, physicians and Infection Preventionists (IPs).

Measures initiated include: creation of a central line cart; standardizing practices using competency checklist; engaging the empowered staff to stop any unsafe practices and enforcing aseptic technique; shifting from scrubbing the hub to using an alcohol cap; and adding daily maintenance to the central line bundle component.

## Results

From the 2^nd^ quarter of 2010 to the 1^st^ quarter of 2013, the PICU CLABSI Team monitored 4,792 central line days. For the 3 quarters of 2010 the CLABSI rate ranges from 3.3 to 3.6. A steady decline was noted in 2011 when it reached the lowest rate at 1.6. Yet it rose sharply in the 2^nd^ quarter of 2012 to a rate of 4.8. Most of the positive cases occurred 5 days post insertion. Lastly, after the implementation of the interventions, the rate over the last 2 quarters (4^th^ quarter of 2012 and 1^st^ quarter of 2013) dropped to zero.

## Conclusion

Our team approach effort was associated with a sharp decline in CLABSI rate to zero for two quarters, yet further surveillance studies need to be conducted to evaluate if the team’s effort can sustain zero CLABSI rate for a long time.

## Disclosure of interest

None declared.

